# Atrial Strain and Strain Rate: A Novel Method for the Evaluation of Atrial Stunning

**DOI:** 10.5935/abc.20160131

**Published:** 2016-10

**Authors:** Hakan Ozkan, Suleyman Binici, Erhan Tenekecioglu, Hasan Ari, Tahsin Bozat

**Affiliations:** 1 Bahcesehir University Faculty of Medicine - Department of Cardiology, Istanbul - Turquia; 2 Ortadogu Hospital - Department of Cardiology, Adana - Turquia; 3 Yuksek Ihtisas Education and Research Center - Department of Cardiology, Bursa - Turquia; 4 Medicalpark Hospital - Department of Cardiology, Bursa - Turquia

**Keywords:** Atrial Fibrillation, Arrhythmia, Cardiac, Myocardial Stunning, Echocardiography, Transesophageal, Electric Cardioversion

## Abstract

**Background:**

Atrial fibrillation (AF) is the most common arrhythmia seen in adults. Atrial stunning is defined as the temporary mechanical dysfunction of the atrial appendage developing after AF has returned to sinus rhythm (SR).

**Objectives:**

We aimed to evaluate atrial contractile functions by strain and strain rate in patients with AF, following pharmacological and electrical cardioversion and to compare it with conventional methods.

**Methods:**

This study included 41 patients with persistent AF and 35 age-matched control cases with SR. All the AF patients included in the study had transthoracic and transesophageal echocardiography performed before and after. Septum (SEPsSR), left atrium (LAsSR) and right atrium peak systolic strain rate (RAsSR) were defined as the maximum negative value during atrial contraction and septum (SEPε), left atrium (LAε) and right atrium peak systolic strain (RAε) was defined as the percentage of change. Parameters of two groups were compared.

**Results:**

In the AF group, 1st hour and 24th hour LAε, RAε, SEPε, LAsSR, RAsSR, SEPsSR found to be significantly lower than in the control group (LAε: 2.61%±0.13, 3.06%±0.19 vs 6.45%±0.27, p<0.0001; RAε: 4.03%±0.38, 4.50%±0.47 vs 10.12%±0.64, p<0.0001; SEPε: 3.0%±0.22, 3.19%±0.15 vs 6.23%±0.49, p<0.0001; LAsSR: 0.61±0.04s-^1^, 0.75±0.04s-^1^ vs 1.35±0.04s-^1^, p<0.0001; RAsSR: 1.13±0.06s-^1^, 1.23±0.07s-^1^ vs 2.10±0.08s- ^1^, p<0.0001; SEPsSR: 0.76±0.04s- ^1^, 0.78±0.04s- ^1^ vs 1.42±0.06 s- ^1^, p<0.0001).

**Conclusion:**

Atrial strain and strain rate parameters are superior to conventional echocardiographic parameters for the evaluation of atrial stunning in AF cases where SR has been achieved.

## Introduction

Atrial fibrillation (AF) is the most common arrhythmia seen in adults. Its incidence increases with age, and the total length of hospitalization is the longest of all the arrhythmias.^1^ Atrial stunning is defined as the temporary mechanical dysfunction of the atrial appendage developing after AF has returned to sinus rhythm (SR) by cardioversion.^2^ Although SR is obtained with an electrocardiograph in clinical practice, the risk of thromboembolic events development increases within hours or weeks, due to atrial appendage contractile dysfunction.^3^ Atrial stunning has been evaluated in studies conducted with transthoracic and transesophageal echocardiography (TTE and TEE, respectively) by measuring diastolic flow velocities, velocity-time integrals, atrial ejection force, lateral annulus tissue Doppler velocities, spontaneous echo contrast (SEC) and atrial flow velocities. In our study, we aimed to evaluate atrial contractile functions by strain and strain rate, which is a new echocardiographic evaluation method in patients with AF, following pharmacological and electrical cardioversion and compare them with conventional methods.

## Methods

We performed 45 elective echocardiographic evaluations. This study consisted of 41 of those 45 participants (15 male and 26 female), whose SR was achieved with persistent AF and who applied to the cardiology department, and 35 control group cases (14 male and 21 female) with SR and no severe valve disease on echocardiography. The study protocol was approved by the local ethics committee. Written informed consent was obtained before enrollment. The cases with pre-procedure contraindications for oral anticoagulation, New York Heart Association class III-IV congestive heart failure, severe native valve and prosthetic valve disease, left atrial thrombus detected in TEE, sick sinus syndrome and hyperthyroidism, and left atrial diameter larger than 5.5 cm measured with TTE were excluded from the study. All the AF patients included in the study had TTE and TEE performed before cardioversion, as recommended by the American Society of Echocardiography. After SR was provided, they had conventional echocardiographic evaluations with TTE at the 1^st^ hour, 24^th^ hour and 1^st^ month controls, and strain and strain rate examinations were evaluated. The Vivid 7 Pro was used for echocardiographic evaluations, the 3.5-MHz probe for TTE and the multiplane 6-MHz probe was used for TEE. Left ventricle ejection fraction was measured by the modified Simpson method. The sampling volume was placed parallel to the ventricle filling flow at the apical four-chamber plan between the mitral and tricuspid valve endings, the peak early diastolic flow velocity (E), the peak late diastolic flow velocity (A), the peak early and late diastolic flow velocity ratios (E/A) for pulsed Doppler evaluation and velocity-time integrals of peak early and late diastolic flow velocities, the left atrial ejection force [0.5×1.06×mitral valve area×(peak A velocity)^2^ Kdyne] and the right atrial ejection force [0.5×1.06×tricuspid valve area×(peak A velocity)^2^ Kdyne] were calculated and atrial functions were evaluated by conventional methods.

In the strain rate echocardiographic apical four-chamber evaluation, in an average 158 number of frames, the peak systolic strain and peak systolic strain rates were measured from the left atrium, the right atrium and the inter atrial septum 5mm above the atrioventricular junction. All values were found based on the average of four consecutive cycles. Peak septum systolic strain rate (SEPsSR), peak left atrium systolic strain rate (LAsSR) and peak right atrium systolic strain rate (RAsSR) were defined as the maximum negative value during atrial contraction and peak septum systolic strain (SEPε), peak left atrium systolic strain (LAε) and peak right atrium systolic strain (RAε) were defined as the percentage of change. After being recorded on digital media, the images were analyzed offline in the EchoPac PC (GE Vingmed Ultrasound) program. Before cardioversion, AF cases were given aspirin and intravenous standard heparin infusion (17 u/kg) and the activated partial thromboplastin time was adjusted to remain at 1.5-2.0 times its value. After TTE and TEE, the cases without intracardiac thrombus were randomly divided into two groups to receive either medical cardioversion with amiodarone infusion (5 mg/kg intravenous loading dose, continuous infusion totaling 1.2 gr) or transthoracic direct current cardioversion. For cases where SR was achieved, anticoagulation with warfarin was provided, keeping international normalized ratio (INR) values at 2.0-3.0. All other antiarrhythmic medications and digoxin were stopped before cardioversion, while other medications were continued. Intravenous midazolam was used for sedation. Transthoracic electrical direct current cardioversion was performed by using synchronized monophasic direct current with the cardioverter defibrillator device. For cardioversion, in order, 200 joules, 300 joules and 360 joules of energy were used. SR was achieved with electrical cardioversion in cases where SR could not be medically provided.

### Statistical evaluation

Was performed with the SPSS 10.0 program. Continuous variables are presented as mean ± standard deviation, whereas categorical variables are presented as percentages. Categorical variables were evaluated by the Pearson chi-square or Fisher's exact chi-square test, and continuous variables were evaluated by the unpaired Student *t* test or Mann-Whitney U test. For all evaluations, a p value < 0.05 was accepted as statistically significant.

## Results

The results from the 41 patients, among the performed 45 elective echocardiographic evaluations, where SR was achieved, were compared to those of the control group. The comparison of demographic characteristics of the two groups is demonstrated in Table 1.

**Table 1:** Baseline characteristics

	AF group (n=41)	Controlgroup (n=35)	p value
Age (year)	59.38±1.40	55.83±1.65	NS
Male	15 (36.59%)	14 (%40)	NS
Female	26 (64.41%)	21 (%60)	NS
AF duration (day)	207.76±47.72	-	
Baseline heart rate (bpm)	94.29±2.21	72.57±1.81	0.001
Body surface area (m^2^)	1.84±0.27	1.79±0.13	NS
Systolic blood pressure (mm Hg)	125.67±2.88	128.00 ±3.05	NS
Diastolic blood pressure (mm Hg)	79.44±1.54	79.57±1.88	NS
Diabetes mellitus	7 (15.6%)	11 (21.4%)	NS
Hypertension	25 (55.6%)	15 (42.9%)	NS
Dyslipidemia	6 (13.3%)	2 (5.7%)	NS
CAD	5 (11.1%)	7 (20%)	NS
Smoking	17 (37.8%)	11 (21.4%)	NS
Acetylsalicylic acid	36 (8%0)	12 (34.3%)	<0.0001
Beta-blockers	13 (28.9%)	9 (25.7%)	NS
Calcium channel blockers	10 (22.2%)	10 (28.6%)	NS
Nitrate	4 (8.9%)	3 (8.6%)	NS
Digoxin	15 (33.3%)	1 (2.9%)	0.001
ACEI	14 (31.1%)	8 (22.9%)	NS
ARB	5 (11.1%)	4 (11.4%)	NS
Lipid lowering drugs	6 (13.3%)	2 (5.7%)	NS
Diuretics	11 (24.4%)	4 (11.4%)	NS
Warfarin	3 (6.7%)	-	NS

AF: atrial fibrillation; CAD: coronary artery disease; ACEI: angiotensin-converting enzyme inhibitors; ARB: angiotensin receptor blocker; NS: non-significant; p > 0.05.

The duration of AF was evaluated by patient history. The AF duration was estimated according to the first diagnosed AF ECG date. The mean AF duration in SR achieved cases was 207.76±47.72 days. The mean heart rate in the AF group before the procedure (94.29±2.21 bpm) was significantly higher than in the control group (72.57±1.81 bpm, p=0.001) (Table 1). Baseline echocardiographic parameters are shown in Table 2.

**Table 2:** Baseline echocardiographic comparison of two groups

	AF group (n=41)	Control group (n=35)	p value
Left atrium (cm)	4.47±0.06	4.37±0.06	NS
LVEDD (cm)	4.80±0.10	4.81±0.97	NS
LVESD (cm)	3.27±0.11	3.08±0.10	NS
Septum (cm)	1.15±0.03	1.14±0.03	NS
Posterior wall (cm)	1.13±0.03	1.06±0.03	NS
Ejection fraction (%)	61.08±1.63	64.14±1.59	NS
Fractional shortening (%)	32.09±1.16	34.85±1.08	NS
End-systolic volume (mL)	45.44±4.34	40.22±3.41	NS
End-diastolic volume (mL)	105.52±6.27	101.31±5.00	NS
Pulmonary artery pressure (mmHg)	27.51±0.93	27.17±1.06	NS
**Mitral regurgitation**			
None	5 (11.1%)	9 (25.7%)	NS
Mild	37 (82.2%	9 (25.7%)	NS
Moderate	3 (6.7%)	3 (8.6%)	NS
**Aortic regurgitation**			
None	12 (26.7%)	13 (37.1%)	NS
Mild	30 (66.7%)	21 (60%)	NS
Moderate	3 (6.7%)	1 (2.9%)	NS
**Tricuspid regurgitation**			
None	32 (71.1%)	23 (65.7%)	NS
Mild	12 (26.7%)	12 (34.3%)	NS
Moderate		1 (2.2%)	NS
LAA peak emptying velocity (cm/s)	37.05±1.73	-	
LAA mean emptying velocity (cm/s)	29.82±1.45	-	
LAA peak filling velocity (cm/s)	36.68±1.77	-	
LAA mean filling velocity (cm/s)	29.60±1.41	-	

AF: atrial fibrillation; LVESD: left ventricle end-systolic diameter; LVEDD: left ventricle end-diastolic diameter; LAA: left atrial appendage; NS: non-significant; p > 0.05.

Sinus rhythm was obtained in 41 patients (91.1%) following cardioversion. AF recurrence was observed in one patient 7 hours after the procedure, and in one patient 10 hours after the procedure. At the end of the first month, a total of 32 patients (71.1%) had SR. The atrial strain and atrial strain rate values of patients who had achieved and remained in SR were compared to the control group. In the AF group, 1^st^ hour (2.61%±0.13) and 24^th^ hour (3.06%±0.19) LA systolic strain were found to be significantly lower than in the control group (6.45%±0.27) (p<0.0001). At the end of the first month, there was no significant difference between two groups. In the AF group, 1^st^ hour (0.61±0.04s^-1^) and 24^th^ hour (0.75±0.04s^-1^) LA peak systolic strain rates were found to be significantly lower than in the control group (1.35±0.04s^-1^) (p<0.0001). There was no statistically significant difference at the end of the first month. In the AF group, SEP systolic strains of the 1^st^ hour (3.0%±0.22) and of the 24^th^ hour (3.19%±0.15) were found to be significantly lower than in the control group (6.23%±0.49) (p<0.0001). Similarly, there was no statistically significant difference at the end of first month. In the AF group, the SEP peak systolic strain rates at the 1^st^ hour (0.76±0.04s ^-1^) and 24^th^ hour (0.78±0.04s^-1^) were found to be significantly lower than in the control group (1.42±0.06s^-1^) (p<0.0001). At the end of the first month, there was no statistically significant difference between two groups. In the AF group, RA systolic strains of the 1^st^ hour (4.03%±0.38) and of the 24^th^ hour (4.50%±0.47) were found to be significantly lower than in the control group (10.12%±0.64) (p<0.0001). In the AF group, the RA peak systolic strain rates at the 1^st^ hour (1.13±0.06s^-1^) and 24^th^ hour (1.23±0.07 s^-1^) were found to be significantly lower than in the control group (2.10±0.08s^-1^) (p<0.0001). At the end of the first month, there was no significant difference in the RA peak systolic strain rate of the AF group (2.07±0.10s^-1^) when compared to that of the control group (2.10±0.08s^-1^).

The flow rates through the mitral and tricuspid valve were evaluated in the AF group and compared with the control group. The findings are shown in Tables 3 and 4.

**Table 3:** Comparison of conventional echocardiographic parameters of left atrium functions between two groups after cardioversion

	AF group	Control group	p value
**Mitral E velocity (m/s)**			
1^st^ hour	0.82±0.04	0.83±0.02	NS
24^th^ hour	0.80±0.04	0.83±0.02	NS
1^st^ month	0.81±0.04	0.83±0.02	NS
**Mitral A velocity (m/s)**			
1^st^ hour	0.61±0.04	0.79±0.02	0.016
24^th^ hour	0.64±0.03	0.79±0.02	0.029
1^st^ month	0.79±0.04	0.79±0.02	NS
**Mitral E/A ratio**			
1^st^ hour	1.34±0.10	1.16±0.04	0.004
24^th^ hour	1.30±0.08	1.16±0.04	0.018
1^st^ month	1.10±0.04	1.16±0.04	NS
**Mitral A time-velocity integral**			
1^st^ hour	5.84±0.38	6.20±0.17	NS
24^th^ hour	6.12±0.41	6.20±0.17	NS
1^st^ month	6.56±0.51	6.20±0.17	NS
**Left atrial ejection force (kdyne)**			
1^st^ hour	8.23±0.96	11.82±0.61	0.015
24^th^ hour	9.36±0.99	11.82±0.61	0.043
1^st^ month	11.67±1.01	11.82±0.61	NS

AF: atrial fibrillation; NS: non-significant; p >0.05.

**Table 4:** Comparison of conventional echocardiographic parameters of right atrium functions between two groups after cardioversion

	AF	Control	p value
**TV E velocity (m/s)**			
1^st^ hour	0,52±0,01	0,58±0,02	NS
24^th^ hour	0,54±0,01	0,58±0,02	NS
1^st^ month	0,55±0,02	0,58±0,02	NS
**TV A velocity (m/s)**			
1^st^ hour	0,36±0,01	0,44±0,01	0,04
24^th^ hour	0,40±0,01	0,44±0,01	NS
1^st^ month	0,45±0,01	0,44±0,01	NS
**TV E/A ratio**			
1^st^ hour	1,36±0,04	1,26±0,06	NS
24^th^ hour	1,35±0,03	1,26±0,06	NS
1^st^ month	1,22±0,03	1,26±0,06	NS
**TV A time-velocity integral**			
1^st^ hour	3,86±0,19	4,72±0,24	NS
24^th^ hour	4,21±0,21	4,72±0,24	NS
1^st^ month	4,66±0,23	4,72±0,24	NS
**Right atrial ejection force (kdyne)**			
1^st^ hour	3,03±0,21	5,88±0,43	0,010
24^th^ hour	3,28±0,21	5,88±0,43	0,023
1^st^ month	5,45±0,36	5,88±0,43	NS

AF: atrial fibrillation; TV: tricuspid valve; NS: non-significant; p > 0.05.

In the subgroup analysis of the study, patients who underwent cardioversion due to AF were divided into two groups according to AF durations as less than 1 year (Group A) and more than 1 year (Group B). These groups were compared amongst themselves and the control group (Group C). There were 24 patients in Group A with a mean age of 57.63±2.13 years, 17 patients in Group B with a mean age of 61.38±1.70 years, and there were 35 patients in Group C with a mean age of 55.83±1.65 years. There was no statistically significant difference between the groups in terms of risk factors, body surface area, left atrial diameter, left ventricle size, left ventricle volumes, left ventricle ejection fraction and fractional shortening and valve insufficiencies.

When the patients in Groups A and B, who achieved SR after a successful cardioversion were compared, while left atrial systolic strain and right atrial systolic strain, left atrial peak systolic strain rate and right atrial peak systolic strain rate were similar in the 1^st^ and 24^th^ hour, they were significantly lower in Group B at the end of the 1^st^ month (6.38%±0.45 and 4.46%±0.34 p=0.01, 11.66%±1.22 and 6.88%±0.32 p=0.02, 1.53±0.13 s^-1^and 1.05±0.03 s^-1^ p=0.02, 2.22±0.08 s^-1^and 1.78±0.23 s^-1^ p=0.04, respectively). There was no significant difference between the two groups with respects to SEP systolic strain and SEP peak systolic strain rate. When the values of Group B were compared to Group C, the left atrial systolic strain and right atrial systolic strain, left atrial peak systolic strain rates and right atrial peak systolic strain rates of Group B were found to be significantly lower (4.46%±0.34 and 6.45%±0.27 p=0.03, 6.88%±0.32 and 10.12%±0.64 p=0.02, 1.05±0.03 s^-1^ and 1.35±0.04 s^-1^ p=0.03, 1.78±0.23 s^-1^ and 2.10±0.08 s^-1^ p=0.04, respectively). There was no statistically significant difference found in Group A and Group C at the end of the 1^st^ month in terms of strain and strain rate parameters (Table 5).

**Table 5:** Comparison of atrial strain and strain rate parameters according to AF duration

	Group A (n=24)	Group B (n=17)	Group C (n=35)		p value	
				A-B	A-C	B-C
**LAε (%)**						
1^st^ hour	2.72±0.13	2.45±0.25	6.45±0.27	NS	<0.0001	<0.0001
24^th^ hour	3.09±0.24	3.02±0.34	6.45±0.27	NS	<0.0001	<0.0001
1^st^ month	6.38±0.45	4.46±0.34	6.45±0.27	0.01	NS	0.03
**SEPε (%)**						
1^st^ hour	3.21±0.36	2.75±0.22	6.23±0.49	NS	<0.0001	<0.0001
24^th^ hour	3.17±0.18	3.22±0.26	6.23±0.49	NS	<0.0001	<0.0001
1^st^ month	6.17±0.67	5.79±0.60	6.23±0.49	NS	NS	NS
**RAε (%)**		w				
1^st^ hour	3.80±0.33	3.31±0.33	10.12±0.64	NS	<0.0001	<0.0001
24^th^ hour	5.10±0.70	4.51±0.32	10.12±0.64	NS	<0.0001	<0.0001
1^st^ month	11.66±1.22	6.88 ± 0.32	10.12±0.64	0.02	NS	0.02
**LAsSR(s¯^1^)**						
1^st^ hour	0.67±0.06	0.53±0.06	1.35±0.04	NS	<0.0001	<0.0001
24^th^ hour	0.86±0.07	0.59±0.06	1.35±0.04	0.013	<0.0001	<0.0001
1^st^ month	1.53±0.13	1.05±0.03	1.35±0.04	0.02	NS	0.03
**SEPsSR (s¯^1^)**						
1^st^ hour	0.83±0.05	0.67±0.07	1.42±0.04	NS	<0.0001	<0.0001
24^th^ hour	0.83±0.05	0.70±0.07	1.42±0.04	NS	<0.0001	<0.0001
1^st^ month	1.35±0.09	1.28±0.11	1.42±0.04	NS	NS	NS
**RAsSR (s¯^1^)**						
1^st^ hour	1.16±0.07	1.08±0.13	2.10±0.08	NS	<0.0001	<0.0001
24^th^ hour	1.32±0.09	1.08±0.14	2.10±0.08	NS	<0.0001	<0.0001
1^st^ month	2.22±0.08	1.78±0.23	2.10±0.08	0.04	NS	0.04

AF: atrial fibrillation; LAε: peak left atrium systolic strain; SEPε: peak septum systolic strain; RAε: peak right atrium systolic strain; LAsSR: peak left atrium systolic strain rate; SEPsSR: peak septum systolic strain rate; RAsSR: peak right atrium systolic strain rate; NS: non-significant; p >0.05.

When the conventional echocardiographic parameters were evaluated, there was no statistically significant difference between the three groups at the end of the 1^st^ month for mitral and tricuspid valves A wave flow velocities, velocity-time integrals, diastolic E/A ratios and atrial ejection force measurements (Table 6).

**Table 6:** Comparison of conventional parameters of left and right atrium functions according to AF duration

	Group A (n=24)	Group B (n=17)	Group C (n=35)		p value	
				A-B	A-C	B-C
**Left atrial ejection force (kdyne)**						
1^st^ hour	10.05±1.40	5.50±0.83	12.40±0.66	0.019	NS	<0.0001
24^th^ hour	10.98±1.39	6.59±0.92	12.40±0.66	0.032	NS	<0.0001
1^st^ month	12.32±1.16	10.63±1.42	12.40±0.66	NS	NS	NS
**Mitral A velocity (m/s)**						
1^st^ hour	0.73±0.05	0.45±0.07	0.79±0.04	<0.0001	NS	<0.0001
24^th^ hour	0.69±0.04	0.51±0.07	0.79±0.04	0.04	NS	<0.0001
1^st^ month	0.76±0.06	0.68±0.05	0.79±0.04	NS	NS	NS
**Mitral A time-velocity integral**						
1^st^ hour	6.73±0.56	4.58±0.25	7.20±0.17	0.004	NS	<0.0001
24^th^ hour	6.94±0.59	4.80±0.27	7.20±0.17	0.01	NS	<0.0001
1^st^ month	7.18±1.08	7.17±0.97	7.20±0.17	NS	NS	NS
**Mitral E/A ratio**						
1^st^ hour	1.24±0.08	2.05±0.15	1.16±0.08	<0.0001	NS	<0.0001
24^th^ hour	1.18±0.06	1.73±0.12	1.16±0.08	0.004	NS	0.001
1^st^ month	1.12±0.05	1.32±0.13	1.16±0.08	NS	NS	NS
**Right atrial ejection force (kdyne)**						
1^st^ hour	3.11±0.24	2.91±0.40	4.88±0.11	NS	0.016	0.0001
24^th^ hour	3.33±0.20	3.21±0.45	4.88±0.11	NS	NS	0.001
1^st^ month	4.68±0.39	4.40±0.70	4.88±0.11	NS	NS	NS
**TV A velocity (m/s)**						
1^st^ hour	0.42±0.01	0.33±0.01	0.44±0.01	0.005	NS	0.0001
24^th^ hour	0.42±0.01	0.35±0.01	0.44±0.01	0.023	NS	0.005
1^st^ month	0.45±0.01	0.41±0.02	0.44±0.01	NS	NS	NS
**TV A time-velocity integral**						
1^st^ hour	4.40±0.22	3.76±0.19	4.72±0.24	NS	NS	NS
24^th^ hour	4.49±0.23	3.91±0.20	4.72±0.24	NS	NS	NS
1^st^ month	4.67±0.23	4.18±0.23	4.72±0.24	NS	NS	NS
**TV E/A ratio**						
1^st^ hour	1.27±0.03	1.44±0.07	1.26±0.06	NS	NS	NS
24^th^ hour	1.26±0.03	1.31±0.07	1.26±0.06	NS	NS	NS
1^st^ month	1.17±0.04	1.19±0.09	1.26±0.06	NS	NS	NS

AF: atrial fibrillation; TV: tricuspid valve; NS: non-significant; p > 0.05.

## Discussion

One of the primary results in our study was that in the early period, the contractile functions were depressed in both right and left atriums when cases with persistent AF, who had SR achieved following medical or electrical cardioversion, were compared with the control group. With conventional echocardiographic parameters, strain and strain rate examinations, atrial functions were observed to recover at the end of the first month. The second, duration of AF before cardioversion is the most important factor related to the severity of atrial stunning.

Atrial stunning is defined as the transient mechanic dysfunction that develops in the atrium and atrial appendage after SR is achieved by cardioversion in AF cases. The incidence of atrial stunning is reported as being 38%-80%.^2^

Atrial stunning is important in SR achieved patients following cardioversion because it can lead to thrombus formation and prepares a basis for thromboembolic events. The factors determining the duration and severity of atrial stunning include the duration of AF, size of the atrium and presence of underlying structural heart disease. The duration of AF before cardioversion is the most important factor determining the duration and severity of atrial stunning. As the duration of AF increases, the rate of returning back from atrial stunning delays.^4^ Another factor that determines the duration and severity of atrial mechanical dysfunction is the size of the atrium. In conducted studies, associations have been found between left atrial size and atrial stunning. When Mattioli et al.^5^ compared cases with large left atriums with those of normal left atriums, they have demonstrated that in large atriums, atrial stunning was more severe and prolonged. When left atrial appendage flow rates were evaluated with TEE in the study by Omran et al.,^6^ they found that, as the left atrial size increased, the flow rates decreased. In our research, the aim was to evaluate atrial contractile functions and atrial stunning with the new echocardiographic parameters of strain and strain echocardiography in patients who had returned to SR after cardioversion and compare them to conventional echocardiographic parameters.

The duration of AF before cardioversion is the most important factor determining the duration and severity of atrial stunning. As the duration of AF increases, it takes longer to recover from atrial stunning.^5,7-11^ In the study by Manning et al.,^8^ they have demonstrated that atrial stunning recovered immediately in cases where the duration of AF was shorter than two weeks before cardioversion, recovered within 24 hours in cases where duration of AF was between two and six weeks and recovered in one week in cases longer than six weeks. With patients having received pharmacologic and electrical cardioversion, Shapiro et al.^11^ have found no difference in atrial stunning between cases with a less than one week AF duration and the control group in their study of AF. However, they did find significantly lower transmitral flow rates in AF cases with a duration longer than one week when compared to the control group and the less than one week duration AF group.

In the subgroup analysis of our study, we compared the cases of AF duration longer than one year, with the cases with duration less than one year and the control group. In cases where the AF was longer than one year, at the end of the first month, lower strain and strain rate values for the right and left atriums were found compared to cases where AF was shorter than one year and the control group. While significant results have been found in the 1^st^ hour and 24^th^ hour evaluations in terms of conventional echocardiographic parameters for atrial mechanical dysfunction, there was no significant difference found statistically when compared with both control and AF cases of less than one year at the end of the first month. In all cases where SR was achieved, the "p" waves were visualized on ECG after cardioversion, which reflects atrial electrical activity. However, atrial mechanical functions were found to be depressed with conventional echocardiographic parameters, strain and strain rate evaluations.

The "A" wave, which reflects atrial contraction, was demonstrated on the Doppler echocardiography of the mitral and tricuspid flows in all cases in the early period following cardioversion. However, atrial contractile dysfunction was detected with atrial strain and strain rate evaluation. Demonstration of the presence of an "A" wave is not adequate for the evaluation of atrial contractile functions with Doppler echocardiography in cases where SR was achieved following cardioversion. The superiority of strain and strain rate examination over conventional methods can be explained by the direct and quantitative lengthening and shortening of atrial myofibrils. There are limited numbers of studies in literature that evaluate atrial systolic functions with strain and strain rate echocardiographic examination. The study by Inaba et al.^12^ has demonstrated that before the left atrium expands, passive stretching of the atrial wall is compromised in cases with paroxysmal AF, since this pathology cannot be demonstrated with methods of conventional echocardiography, they did demonstrate it in the early period of the disease with strain rate echocardiography. Modesto et al.^13^ have compared conventional echocardiographic parameters with strain and strain rate echocardiography to determine the prevalence of atrial dysfunction in patients with cardiac amyloidosis. As a result of that study, while atrial functions were found to be normal with conventional echocardiographic parameters in patients with cardiac amyloidosis, atrial dysfunction was demonstrated quantitatively in those patients with strain and strain rate echocardiography. The results obtained in our study support the results of those two studies. Furthermore, mitral and tricuspid valve peak A flow rates are affected by various factors, such as ventricle compliance, heart rate, site of the sample taken, and preload and afterload.^14,15^ The other conventional parameters, including E/A ratio, A wave rate-time interval and atrial ejection force, are used to calculate the peak A flow passing the valves. In clinical and experimental studies, structural remodeling leading to fibrosis and atrial cardiomyopathy has been demonstrated in the atrium as a response to atrial arrhythmia. This structural remodeling is associated with the duration of the AF and is a progressive process. Sometimes structural remodeling may not completely return to normal, especially due to the development of atrial fibrosis and atrial cardiomyopathy in cases with long duration of AF.^16^ In one study, atrial mechanical function was deteriorated more severely in cases where AF lasted longer than three years and recovery of mechanical dysfunction lasted longer after cardioversion.^17^ In our study, with strain and strain rate echocardiography, it was demonstrated that atrial stunning continued at the end of the first month in cases with an AF duration longer than one year. Due to the long duration of AF in these cases, the progressive process of structural remodeling affects atrial myocytes more severely and prepares a basis for the development of atrial cardiomyopathy.

### Clinical implication

Atrial strain and strain rate echocardiographic evaluation is a useful method for the evaluation of atrial stunning in AF cases where SR has been achieved. In cases having had cardioversion due to persistent AF, this new method is superior to the A wave measured from the mitral and tricuspid valve with pulse Doppler and the dependent parameters of A wave speed-time integral, E/A rate and atrial ejection force in demonstrating the continuation of atrial stunning. Atrial strain and strain rate echocardiographic examination reflects atrial systolic functions quantitatively, and can be used safely both in the diagnosis phase and in planning optimal medical treatment, including anticoagulant therapy in cases with persistent atrial stunning.

### Study limitation

The main limitations of this study were the relatively small number of patients and the short duration of follow-up. The limited number of patients in this study may not represent the whole population. So, the inclusion of a larger and more representative group of patients would provide a more comprehensive picture. We did not evaluate the diastolic and systolic functions by hemodynamic and biochemical tests. Assessment of the relationship between strain, strain rate and diastolic and systolic functions by hemodynamic and biochemical tests might be more illuminating.

## Conclusion

The results of this pilot study on atrial stunning showed atrial strain and strain rate parameters to be better than conventional echocardiographic parameters in AF cases where SR has been achieved. This result needs to be confirmed with a larger group of patients and long-term follow-up studies.
